# The Prognostic Role of *RASSF1A* Promoter Methylation in Breast Cancer: A Meta-Analysis of Published Data

**DOI:** 10.1371/journal.pone.0036780

**Published:** 2012-05-17

**Authors:** Yong Jiang, Lin Cui, Wen-de Chen, Shi-hai Shen, Li-dong Ding

**Affiliations:** 1 Department of Oncology, Jiangyan People’s Hospital, the Affiliated Hospital of Yangzhou University, Jiangyan, China; 2 Department of Scientific Research, Jiangyan People’s Hospital, the Affiliated Hospital of Yangzhou University, Jiangyan, China; Ohio State University Medical Center, United States of America

## Abstract

**Purpose:**

Epigenetic alterations have been investigated as prognostic indicators in breast cancer but their translation into clinical practice has been impeded by a lack of appropriate validation. We present the results of a meta-analysis of the associations between *RASSF1A* promoter methylation status and both disease free survival (DFS) and overall survival (OS) in female breast cancer.

**Methods:**

Eligible studies were identified through searching the PubMed, Web of Science and Embase databases. Studies were pooled and summary hazard ratios (HR) with corresponding confidence intervals (CIs) were calculated. Funnel plots were also carried out to evaluate publication bias.

**Results:**

A total of 1795 patients from eight studies were included in the meta-analysis. There are eight studies which investigated DFS in 1795 cases. The relative hazard estimates ranged from 1.77–5.64 with a combined HR of 2.75 (95%CI 1.96–3.84). The HR of *RASSF1A* promoter methylation on DFS adjusted for other potential prognostic factors was 2.54 (95%CI 1.77–3.66). There has been five trials which analyzed the associations of *RASSF1A* promoter methylation status with OS in 1439 patients. The hazard estimates ranged from 1.21–6.90 with a combined random-effects estimates of 3.47 (95%CI 1.44–8.34). OS reported in multivariate analysis was evaluated in four series comprising 1346 cases and the summarized random-effects HR estimate was 3.35 (95%CI 1.14–9.85). Additionally, no publication bias was detected for both OS and DFS.

**Conclusion:**

The results of this meta-analysis suggest that *RASSF1A* promoter hypermethylation confers a higher risk of relapse and a worse survival in patients with breast cancer. Large prospective studies are now needed to establish the clinical utility of *RASSF1A* promoter methylation.

## Introduction

Breast cancer (BC) is the most commonly diagnosed cancer and the leading cause of cancer death in females worldwide, accounting for 23% (1.38 million) of the total new cancer cases and 14% (458,400) of the total cancer deaths in 2008 [1]. Because of early detection and effective adjuvant medical treatments, the survival rate of breast cancer has increased during the past decades. However, breast cancer is remarkably heterogeneous in histology and genetics, as well as in clinical behavior. Traditionally, pathologic determinations of tumor size, lymph node status, endocrine receptor status, histological grade, and human epidermal growth factor receptor 2 (HER2) expression have driven prognostic predictions and, ultimately, adjuvant therapy recommendations for patients with breast cancer [2]. Nonetheless, these prognostic and predictive factors are relatively crude measures and it poses a great challenge for clinicians regarding the choice of optimum adjuvant treatment. It is of great importance to avoid overtreatment in patients who only receive a modest benefit, while suffering from more toxic side effects. On the other hand, undertreatment or incorrect treatment has to be avoided as well. Although the current well-established clinical and histological factors and some other well-defined biological factors (e.g., hormone receptors and HER2 status) have been established and are assessed routinely in therapy decision-making and evaluating the prognosis, there are increasing concerns that these prognostic determinants are limited in their ability to capture the diversity of clinical behaviors of breast cancer and that they would be insufficient to predict the response to specific treatment strategies for individual patients. Recently, gene-expression-based prognostic assays are being used to predict breast cancer outcomes, but their prognostic validities are still undergoing evaluation [3]. Therefore, research efforts continue to focus on identifying more sensitive and specific indicators that could more reliably predict clinical outcomes and enhance treatment options.

Bulks of epidemiological and experimental studies have verified epigenetic and genetic changes involved in the development and progression of breast cancer (see review [4]). Recently, changes in the status of DNA methylation, known as epigenetic alterations, have turned out to be one of the most common molecular alterations in human malignancies, including breast cancer [5]. Several potential tumor suppressor genes have been described as frequently silenced by hypermethylation in breast cancer. Among which, RAS-association domain family 1 (*RASSF1A*) is widely investigated. *RASSF1A* (http://www.ncbi.nlm.nih.gov/epigenomics/view/genome/56289?term=Rassf1), which is located at 3p21.3, is functionally involved in cell cycle control, microtubule stabilization, cellular adhesion, motility, and apoptosis [6]. Depletion of *RASSF1A* is reported to be associated with accelerated mitotic progression, an elevated risk for chromosomal defects, enhanced cellular motility, and increased tumor susceptibility in knockout mice [7], [8], [9]. Epigenetic inactivation of *RASSF1A* by hypermethylation of CpG islands in the promoter region [NC_000075.5 (107,453,580–107,454,373)] is observed in a considerable proportion of cancers and is associated with clinicopathological factors in various types of cancers, including breast cancer (see review [10]). Furthermore, *RASSF1A* promoter hypermethylation was reported as a prognostic indicator in renal cell carcinoma, non-small cell lung cancer, neuroblastoma, melanoma, endometrial cancer and breast cancer [11]–[18]. All of these findings suggested that it might play a pivotal role in the development of human cancer.

Despite a number of individual studies performed in breast cancer patients, the prognostic value of *RASSF1A* promoter methylation status in breast cancer patient’s survival remains controversial. Therefore, we performed a systematic review of the literature with meta-analysis to obtain a more accurate evaluation of its prognostic value in breast cancer.

## Results

### Study Selection and Characteristics

Fifty-eight relevant citations were identified for initial review using search strategies as described previously. Of these, forty-six were initially excluded after read the titles and abstracts (13 not about breast cancer; 7 on cell lines; 11 review articles; 10 were on tumor biological behavior; 5 with other gene methylation). Investigators retrieved the remaining 12 citations for full text evaluation. Upon further review, three articles were eliminated on the basis of inadequate data for meta-analysis. Moreover, one was excluded for overlapping publication [19]. Ultimately, the systematic literature search yielded a total of 8 studies comprising 1795 patients for final analysis [15]–[18], [20]–[24].

The characteristics of retained 8 studies are listed in Table 1. The sample size of the included studies ranged from 78 to 670 patients (median sample size, 224 patients). The trials were conducted in 7 countries (Portugal, USA, Saudi Arabia, Tunisia, India, Greece, and Austria) and published between 2005 and 2011. There was 60.9% of BC patients had the methylated *RASSF1A* allele with a frequency ranging from 19.6 to 87.0% (median, 64.0%) in individual trials. The methylated *RASSF1A* levels were detected using either methylation specific PCR (MSP) [18], [22], [23] or quantitative methylation specific PCR (QMSP) [15], [17], [20], [21], [24]. The corresponding primer sequences of PCR are provided in a supplementary table (Table S1). DNA methylation status of *RASSF1A* promoter was assessed in plasma or tumor tissues. Except for one study that used fine-needle aspirate washings [15]. A HR on DFS and OS could be extracted from 5 and 8 of the studies, respectively. Most of the survival data for breast cancer were available in the form of multivariate analysis except for one study reported in univariate form (Kaplan–Meier survival curve) [18].

**Table 1:** Baseline characteristics of eligible studies evaluating RASSF1A hypermethylation and OS or DFS in breast cancer patients.

First Author	Year	Country	Methods	M/N (%)	N	Stage	Grade	Materials	OS	DFS
									HR (95%CI)	HR (95%CI)
Martins [15]	2011	Portugal	QMSP	86	178	0–IV	1–3	fine-needle aspirate washings	NA	2.53 (1.09–5.87)
Cho [17]	2011	USA	QMSP	85.2	670	I–IV	NA	formalin fixed paraffin-embedded tissues	1.21 (0.76–1.93)	1.77 (0.86–3.67)
Gobel [20]	2011	Austria	QMSP	21.8	428	0–IV	1–3	peripheral blood-plasma	5.60 (2.10–14.50)	3.40 (1.60–7.30)
Kioulafa [18]	2009	Greece	MSP	57	93	I–II	1–3	formalin fixed paraffin-embedded tissues	4.31 (0.92–7.58)	3.47 (1.24–9.32)
Buhmeida [21]	2011	Saudi Arabia	QMSP	65	100	I–IV	1–3	formalin fixed paraffin-embedded tissues	NA	5.64 (1.23–25.81)
Karray-Chouayekh [22]	2010	Tunisia	MSP	87	78	I–IV	1–3	fresh-frozen specimens	NA	7.33 (1.37–37.72)
Sharma [23]	2009	India	MSP	63	100	I–III	NA	formalin fixed paraffin-embedded tissues	4.05 (0.47–34.92)	1.80 (0.79–4.09)
Fiegl [24]	2005	Austria	QMSP	19.6	148	I–III	1–3	peripheral blood-plasma	6.90 (1.90–25.90)	5.10 (1.30–19.80)

FFPE, formalin fixed paraffin-embedded; PBP, peripheral blood-plasma; FF, fresh-frozen; FNAW, fine-needle aspirate washings; MSP, methylation specific PCR; QMSP, quantitative methylation specific PCR.

### Meta-analysis

**Figure 1:**
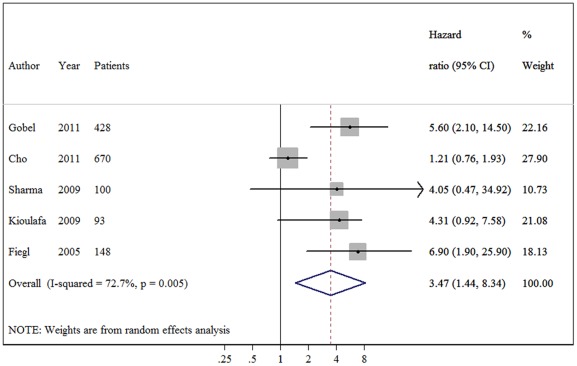
Forest plot showing the association between *RASSF1A* methylation and overall survival (OS) of breast cancer. The summary HR and 95% CIs were shown (according to the random-effects estimations).

**Figure 2:**
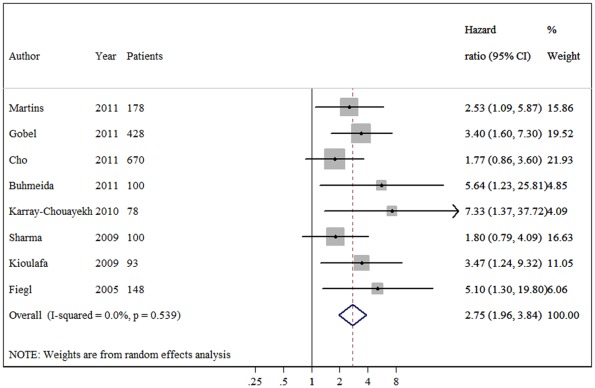
Forest plot showing the association between *RASSF1A* methylation and disease-free survival (DFS) of breast cancer. The summary HR and 95% CIs were shown (according to the fixed-effects estimations).

**Figure 3:**
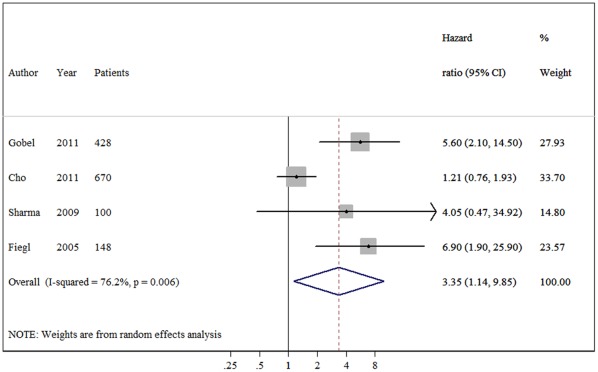
Forest plot showing the association between *RASSF1A* methylation and overall survival (OS) of breast cancer calculating from the data of multivariate Cox regression analyses.

**Figure 4:**
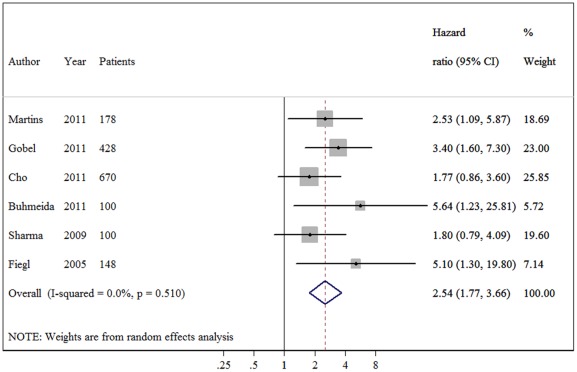
Forest plot showing the association between *RASSF1A* methylation and disease-free survival (DFS) of breast cancer calculating from the data of multivariate Cox regression analyses.

The meta-analysis was carried out for the analyses of all studies on OS, DFS and their subgroups. The main results of the meta-analysis are summarized in Table 2. When all study populations combined, dismal survival outcomes on BC patients with hypermethylation of *RASSF1A* promoter were observed: for overall survival, summary HR = 3.47, 95%CI 1.44–8.34; I^2^ = 72.70%, random-effects model (Figure 1), and for disease free survival, summary HR = 2.75, 95% CI 1.96–3.84; I^2^ = 0.00%, fixed-effects model (Figure 2). Even by carrying out the meta-analysis using the HRs from Cox regression models only, we still observed significant pejorative impacts on OS (HR  = 3.35, 95% CI 1.14–9.85; test for heterogeneity: I^2^ = 76.20%) (Figure 3) and DFS (HR = 2.54, 95% CI 1.77–3.66; test for heterogeneity: I^2^ = 0.00%) (Figure 4). Due to strong heterogeneity existed in the trials aggregated for overall survival, Galbraith plot was used to explore the heterogeneity. The heterogeneity disappeared after omitting one trial by Cho *et al*. (Chi-squared  = 0.38, p  = 0.945) [17].

**Table 2:** Main results of eligible studies evaluating RASSF1A hypermethylation and OS/DFS in breast cancer patients.

	N. of studies/cases	HR (95% CI)	Heterogeneity		
			χ^2^	p	I^2^
**Overall Survival (OS)**					
All studies					
Fixed effects	5/1439	2.10 (1.45–3.03)	14.67	0.005	72.70%
Random effects	5/1439	3.47 (1.44–8.34)	14.67	0.005	72.70%
Cox regression model	4/1346	3.35 (1.14–9.85)	12.63	0.006	76.20%
Testing methods					
QMSP	3/1246	3.28 (0.94–11.50)	12.14	0.002	83.5%
MSP	2/192	4.26 (1.65–10.98)	0.00	0.959	0.00%
Testing materials					
Plasma	2/576	6.03 (2.77–13.11)	0.06	0.801	0.00%
Tissue samples	3/863	2.27 (0.82–6.27)	5.46	0.065	63.4%
**Disease-Free Survival (DFS)**					
All studies					
Fixed effects	8/1795	2.75 (1.96–3.84)	6.01	0.539	0.00%
Cox regression model	6/1624	2.54 (1.77–3.66)	4.28	0.51	0.00%
Testing methods					
QMSP	5/1525	2.77 (1.84–4.15)	3.44	0.487	0.00%
MSP	3/270	2.71 (1.49–4.91)	2.57	0.277	22.1%
Testing materials					
Plasma	2/576	3.74 (1.93–7.26)	0.26	0.610	0.00%
Tissue samples	5/1041	2.54 (1.57–4.13)	4.62	0.328	13.5%

MSP, methylation specific PCR; QMSP, quantitative methylation specific PCR.

In the subgroup analyses on overall survival, a significant prognostic role of *RASSF1A* methylation status was detected in the studies using MSP methods (HR = 4.26, 95%CI 1.65–10.98). However, no statistical significance reached in those using QMSP (HR = 3.28, 95%CI 0.94–11.50). When the differences of material reported for detecting *RASSF1A* promoter methylation levels were taken into consideration, the aggregated survival data showed an unfavorable survival prognosis using plasma (HR 6.03, 95% CI 2.77–13.11), but not tissue samples.

In the subgroup analyses on disease-free survival, a subset of five studies (1525 patients) reporting the DFS for breast cancer patients using QMSP, and a subset of three studies (270 patients) reporting the DFS using MSP were pooled separately. The summary HR estimates for both groups showed inverse correlations with DFS (HR = 2.77, HR = 2.71, respectively). In addition, there was no difference when various materials used in detecting *RASSF1A* methylation status. Furthermore, no evidence of heterogeneity observed in these comparisons. These results suggest that breast cancer patients with *RASSF1A* promoter hypermethylation have a poor prognosis of relapse, irrespective of the detecting methods and samples.

### Assessment of Publication Bias

Visual assessment of the funnel plots provided no evidence of overt publication bias for studies in either of the two outcomes. Further evaluation using Egger’s linear regression test also failed to reveal any evidence for significant publication bias in OS (P = 0.36) and DFS (P = 0.34) study groups.

## Discussion

For proper management of patients with cancer, accurate prognostic and predictive factors are necessary. Such factors are particularly important in breast cancer that has widely varying outcomes and for which systemic adjuvant therapy may be beneficial. Prognostic factors may help us to differentiate those patients with indolent from those with more aggressive disease. Patients with aggressive disease may then be candidates for treatment with systemic adjuvant therapy, while those with indolent disease may be spared the toxic side-effects and costs of this treatment. The accumulating evidence for epigenetic defects in breast cancer may be potentially useful in cancer progression. Aberrant DNA methylation of CpG islands within 5-prime of genes occurs almost in every type of cancer and easy to measure. Potential of gene-specific DNA methylation as a predictor of important clinical features has been explored in a number of studies now. Among which, the tumor suppressor gene *RASSF1A* promoter methylation was reported to be valuable as a prognostic indicator for breast cancer. Due to relatively small samples of individual study and controversial conclusions, we performed this meta-analysis of the literature to analyze whether *RASSF1A* hypermethylation could readily be harnessed as clinically useful predictive biomarker for breast cancer.

This is the first meta-analysis of published studies to evaluate the association between *RASSF1A* promoter methylation and breast cancer prognosis in 1795 cases. Our results using the summarized HR of OS and DFS indicated that hypermethylation of *RASSF1A* is associated with both DFS and OS (pooled HR estimates of 2.75 and 3.47 for DFS and OS, respectively). These effects were slightly attenuated but still significant in multivariate analyses (adjusted HRs of 2.54 and 3.35, respectively), showing that its effect is independent of lymph node status, tumor size and tumor grade as well as a range of other biological variables on multivariate analysis.

When the five studies reported the HR of overall survival were pooled, a considerable degree of interstudy heterogeneity was noticed (I^2^ = 72.7%). We applied Galbraith plot which is visualized in identifying the heterogeneous studies to explore the heterogeneity. When one study by Cho *et al*. was excluded, the hazard size remains significant but the heterogeneity disappeared. The heterogeneity was probably due to the difference in the baseline characteristics of patients (age, tumor stage, race or country), the detecting methods, testing materials, the duration of follow-up or others. For example, when we stratified them according to detecting methods, heterogeneity disappeared in MSP subgroup. Strong heterogeneity still existed in quantitative methylation-specific PCR subgroup. Some techniques features regarding QMSP may partially explain this heterogeneity. First, lack of clear hypermethylation cut-off definition, it should be made about the cut-off value of RASSF1A methylation level for increased survival risk. To date, the researchers use median or self-defined value in their laboratory as the cut-off value and the accurate value was different. In addition, testing materials may also contribute to the heterogeneity, in this subgroup, methylation level detecting using tissue samples was marked (I^2^ = 63.4%). We postulated that the timing from resection to fixation or the process of fixation itself may potentially alter methylation status in paraffin-embedded tumors. One study observed that methylation status varied when different fixation techniques used [25]. We addressed the issue of heterogeneity by a rigorous methodological approach that used a random-effects model for more conservative estimates. Nevertheless, there is no definitive explanation for the heterogeneity.

Some limitations of this meta-analysis should be discussed. First, this analysis was performed at the study level, which limited ability to explore the potential for confounding by various demographic and clinical factors (e.g., ethnicity, hormone receptor status, disease stage, differentiation and treatment regimes). Second, this study was predominately based on the findings of observational studies, which inherently contain greater potential for confounding than randomized controlled trials. Third, potential risk bias was a concern, as published studies are often positive and so the omission of unpublished studies may lead to exaggeration of the summary HR. Although publication bias evaluation did not suggest any bias in the pooled OS and DFS studies, we identified studies only from limited databases, the total number of included studies and the total sample size were relatively small; which might influence the validity of our analysis to some extent. Fourth, the quality of pooled studies influences the level of confidence of meta-analysis remarkably. Published articles often lack sufficient information to allow adequate assessment of the quality of the study or the generalisability of the study results. So REMARK criteria were recommended when reporting tumor markers [26]. Only one involved study reported the prognostic role of *RASSF1A* methylation in BC using REMARK criteria [17]. Finally, most of studies included in the pooled analyses of breast cancer outcomes were carried out in European populations, it is possible that the results of these analyses are not readily generalizable to other populations. Because of these limitations existing in the identified studies and the current meta-analysis, our results should be interpreted with caution and likewise, the conclusions of this meta-analysis should also be drawn carefully.

In conclusion, hypermethylation of *RASSF1A* promoter was found to be independently associated with decreased survival of breast cancer patients. The promoter methylation of the *RASSF1A* gene is potentially useful biomarker for predicting prognosis in breast cancer. Large studies, both observational cohorts and clinical trials, are now urgently needed to test whether hypermethylation of *RASSF1A* can provide prognostic information in addition to currently used standards and also to establish if it has clinical utility.

## Materials and Methods

### Publication Selection

A comprehensive literature search was carried out by two independent reviewers (Jiang Y and Cui L) using the PubMed, Web of Science and Embase databases. The search ended on 9 September 2011. The following keywords were used in various combinations: ‘breast cancer’, ‘biomarkers’, ‘molecular markers’, ‘survival’, ‘prognosis’, ‘RAS-association domain family 1′ and ‘*RASSF1A*’. The search was performed without langue restriction. Reference lists from relevant primary studies and review articles were also checked for additional relevant publications. To be eligible for inclusion, studies had to meet the following criteria: (1) evaluating the association between *RASSF1A* promoter methylation status and the prognosis of breast cancer patients, e.g., disease free survival (DFS) and/or overall survival (OS); (2) hazard ratio (HR) for OS or DFS according to *RASSF1A* methylation status either had to be reported or could be calculated from the data presented; (3) studies should be with full text not only abstracts for relevant information extraction; (4) when the same patient population reported in several publications, only the most recent report or the most complete one was included in this analysis to avoid overlapping between cohorts.

### Definitions and Data Extraction

Overall survival was defined as the interval between the medical treatment (including surgical excision, chemotherapy or radiotherapy) and the death of patients or the last observation. Disease free survival was measured from the date of treatment until the detection of recurrence or the last follow-up assessment. The following data from all eligible publications was extracted respectively by two reviewers (Cui L and Chen WD) with a standardized data extraction form: first author’s surname, year of publication, patient source, sample size, disease stage, tumor grade, methylation status detecting method, positive ratio, and prognostic outcomes of interest (DFS and OS, including the information whether the outcomes were tested by multivariate analysis). Disagreements were resolved by discussion.

### Statistical Analysis

Meta-analysis techniques were used to compute a summary estimate of the hazard ratio (HR) and 95% confidence intervals (CIs) for recurrence or death with breast cancer. Survival outcome data were synthesized using the time-to-event HR as the effective measure. When HR was not provided directly, estimated value was derived indirectly from other presented data using the methods described by Tierney *et al.* [27]. Moreover, when univariate and multivariate analyses of OS and/or DFS were both available, the latter was selected to be combined because survival response variable is influenced by multiple factors. Heterogeneity between the studies was tested using Q-statistics. It was considered statistically significant if p value less than 0.10 and was also quantified using the I^2^ metric (I^2^<25%, no heterogeneity; I^2^ = 25–50%, moderate heterogeneity; and I^2^>50%, strong heterogeneity) [28], [29]. If the heterogeneity was existed, we used a random-effects model in place of a fixed-effects model and the Galbraith plot was used to provide a graphical display to get a visual impression of the amount of heterogeneity from a meta-analysis [30]. By convention, an observed HR>1 implied a worse survival for the group with *RASSF1A* hypermethylation. This impact of *RASSF1A* on survival was considered as statistically significant if the corresponding 95% CI for the summary HR did not overlap 1 unit. Publication bias was assessed by funnel plots and Egger’s linear regression. All p values were two sided. Statistical calculations were all performed using STATA version 11.0, College Station TX.

## Supporting Information

Table S1 **The primer sequences of detecting** ***RASSF1A*** **promoter methylation status of the eligible studies.**
(DOC)
