# Supplementary material for: The Prognostic Role of RASSF1A Promoter Methylation in Breast Cancer: A Meta-Analysis of Published Data
Source: PLoS One. 2012 May 17;7(5):e36780. doi: 10.1371/journal.pone.0036780 (PMC3355150; doi:10.1371/journal.pone.0036780)
Supplement: Table S1 — The primer sequences of detecting RASSF1A promoter methylation status of the eligible studies. (DOC) [file pone.0036780.s001.doc]

| **Table S1** The primer sequences of detecting *RASSF1A* promoter methylation status of the eligible studies. | | | |
| --- | --- | --- | --- |
| Study ID | Method | Forward Primer Sequence | Reverse Primer Sequence |
| Martins[16] | QMSP | GCGTTGAAGTCGGGGTTC | CCCGTACTTCGCTAACTTTAAACG |
| Cho[17] | QMSP | GCGTTGAAGTCGGGGTTC | CCCGTACTTCGCTAACTTTAAACG |
| Gobel[24] | QMSP | ATTGAG TTGCGGGAGTTGGT | ACACGCTCCAACCGAATACG |
| Kioulafa[18] | MSP(Unmethylated) | GGTTGTATTTGGTTGGAGTG | CTACAA ACCTTTACACACAACA |
|  | (Methylated) | GTTGGTATTCGTTGGGCGC | GCACCACGTATACGTAACG |
| Buhmeida[25] | QMSP | ATTGAGTTGCGGGAGTTGGT | ACACGCTCCAACCGAATACG |
| Karray-Chouayekh[26] | MSP(Unmethylated) | TGGTTTTTTTTAGTTTTTTTTTGTT | ACTACCATATAAAATTACACACA |
|  | (Methylated) | GGTTTTTTTTAGTTTTTTTTCGTC | CTACCGTATAAAATTACACGCG |
| Sharma[27] | MSP(Unmethylated) | GGTTGTATTTGGTTGGAGTG | CTACAA ACCTTTACACACAACA |
|  | (Methylated) | GTTGGTATTCGTTGGGCGC | GCACCACGTATACGTAACG |
| Fiegl[28] | QMSP | ATTGAGTTGCGGGAGTTGGT | ACACGCTCCAACCGAATACG |
